# Value-addition of carbohydrate-derived platform molecules using stoichiometric redox reagents

**DOI:** 10.1039/d5ra04323a

**Published:** 2025-08-13

**Authors:** Saikat Dutta

**Affiliations:** a Department of Chemistry, National Institute of Technology Karnataka Surathkal Mangalore-575025 Karnataka India sdutta@nitk.edu.in

## Abstract

Even though redox reactions in organic synthesis are increasingly catalytic in academic and industrial settings, there is still significant scope for stoichiometric redox reagents (SRRs). The works on using SRRs for biorefinery processes and the chemistry of renewables are scattered in the literature. This work reviews the attempts to synthetically transform carbohydrate-derived platform chemicals into fine chemicals using stoichiometric oxidizing and reducing agents. Many stoichiometric oxidants and reductants form relatively harmless byproducts and can be conveniently recovered and recycled. The processes work under reasonably mild conditions without the mandate of using delicate metal-based catalysts and an overpressure of the oxidant. The selectivity, yield, scalability, and process economics involving SRRs can compete with catalytic processes in several instances. The discussion on the reaction mechanisms, selectivity, green metrics, nature of waste, and recyclability of SRRs will help make an informed decision while designing synthetic strategies for the biorefinery processes.

## Abstract 2

The carbohydrate-derived platform chemicals can be transformed into fine chemicals using stoichiometric redox reagents (SRRs). The sustainability parameters and process economics involving SRRs are comparable with catalytic processes in many cases.

## Graphical abstract

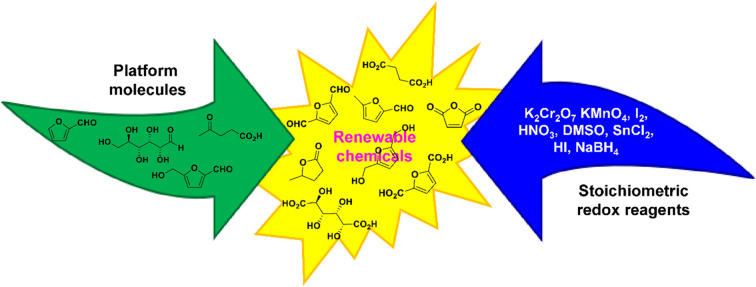

## 1. Introduction

Functional organic molecules are produced in the chemical industries by sequentially introducing various heteroatom-containing functional groups in petroleum-derived hydrocarbons.^1,2^ The oxidation steps have been increasingly catalytic over the past decades for better process economics and eco-friendly character.^3^ However, many SRRs (*i.e.*, oxidants & reductants) continue to be used for downstream multistep synthetic value-addition of the chemical intermediates produced from hydrocarbon.^4^ The advantages of the stoichiometric oxidants include high selectivity, mild reaction conditions, precise control over the kinetics and mechanistic pathway, high stereoselectivity, precise quantification, and high substrate scope.^5,6^ Moreover, SRRs avoid using expensive metal catalysts and the overpressure of gaseous oxidants or reductants. However, SRRs produce equivalent waste as byproducts, which can involve toxic metal salts or gases.^7^ Therefore, if SRRs produce harmless byproducts, the issue of forming significant wastes can be mitigated. For example, when H_2_O_2_ is used as the stoichiometric oxidant, O_2_ and H_2_O are formed as innocuous byproducts. Moreover, the waste issue can be mitigated if the byproducts produced by the SRRs can be recycled in a closed system without releasing them into the environment. For example, the NO_*x*_ formed during the oxidation process involving nitric acid or metal nitrates can be recycled back into forming nitric acid without releasing it into the environment.^8^

The organic chemical manufacturing industries are undergoing a revolution where biogenic carbon is increasingly introduced as a feedstock instead of exclusively relying on exhaustible fossilized carbon.^9^ Using biomass as a renewable carbon source ensures social, economic, and environmental benefits.^10,11^ The biomolecules can be transformed into liquid ‘biofuels’.^12,13^ Alternatively, the biomolecules can also be converted into functionalized organic molecules to produce virtually all classes of organic products of commercial interest (including polymers) that are otherwise sourced from petroleum.^14^ The synthetic transformation of biomolecules into biofuels or fine chemicals involves reduction–oxidation (redox) steps.

The synthetic transformation of biomolecules into functionalized organic chemicals presents various technical challenges.^15^ The biomolecules in abundant biomass are polymeric (*e.g.*, cellulose, lignin), densely functionalized, and heavily oxygenated.^16,17^ The first step of converting biomass into targeted organic molecules often involves a pretreatment process that separates the significant components of biomass, preferably without damaging their native structure.^18^ The purified component is then transformed into a handful of small organic molecules with selected functionalities. These small molecules act as ‘platform chemicals’ to synthesize organic chemicals of desired molecular structure and properties by a series of synthetic steps, often involving redox transformations.^19–21^ The redox steps can benefit from the enormous amount of information in synthetic organic chemistry, assisting in selecting suitable oxidants, catalysts, and process conditions. Innocuous oxidants (*e.g.*, air, O_2_) are commonly used in the presence of an appropriate catalyst for redox chemistry involving biomolecules in biomass feedstock.^22^ In general, stoichiometric redox reagents (SRRs) are more costly and environmentally disadvantageous compared to the redox processes performed under catalytic conditions.^23^ In particular, the SRRs produce a significant volume of wastes that are often toxic and environmental pollutants. Separating and purifying products from the byproducts of SRRs and unreacted SRRs are often challenging and costly. However, due to certain advantages, many stoichiometric oxidants and reductants continue to receive enormous interest. The stoichiometric oxidants are typically more reactive, do not necessitate expensive catalysts, and work under mild conditions. Moreover, their reactivity is predictable, and the stoichiometry can be easily calculated to improve material utilization. Various SRRs have shown remarkable activity and selectivity for the synthetic value-addition of biorenewable chemicals.

Molecular hydrogen (H_2_) is routinely used as the reducing agent for producing various commercial chemicals from biomass.^24^ The processes use an overpressure of H_2_ in the presence of a suitable metal-based catalyst. Even though H_2_ acts as a stoichiometric reducing agent as per the chemical equations, it is used in significant excess compared to the molar requirements, and focus is given to the efficiency of the metal catalyst (*e.g.*, turn-over number, stability, selectivity, recyclability). Catalytic hydrogenation processes of biomass-derived platform chemicals are extensively studied, and these works have been reviewed.^25,26^ These processes are out of the scope of this review. A hydrogen molecule can also be added to an unsaturated molecule *via* a hydrogen donor molecule *via* a catalytic transfer hydrogenation (CTH) reaction.^27^ Molecular hydrogen is produced *in situ*, but the quantity is negligible, to be observed in the gaseous form. Even though a metal-based catalyst candidate is used, the hydrogen donor molecule (HDM) is typically used with equivalent quality or in slight excess. The HDMs get converted into their oxidized form and must be reduced in a different process. For example, when formic acid (FA) or sodium formate is used as HDM, CO_2_ is formed as the byproduct.^28^ Similarly, isopropanol (IPA) is a routinely used HDM for various CTH reactions. The IPA oxidizes to acetone, which must be hydrogenated back to the IPA to replenish.^29,30^ Therefore, these reagents can be considered stoichiometric reducing agents, and some of the recent works in this area are highlighted in this work.

The value-addition of biomass-derived chemicals involving catalytic oxidation and reduction processes has been periodically reviewed.^31,32^ However, much less emphasis is given to works reporting SRRs for such transformations. While catalytic redox processes involving reagents like O_2_ and H_2_ have clear economic and environmental advantages, the challenges associated with these processes are often toned down.^32–36^ These processes require metal-based catalysts, which require elaborate preparation, and the components (*i.e.*, metal, support) are usually expensive and have a significant environmental footprint during their production.^37,38^ The activity and selectivity of the catalyst depend on several parameters, including the particle size of the metal sites, supporting material, solvent, overpressure of the reagent, *etc.*^39^ Therefore, the reproducibility of the catalytic processes requires a high degree of uniformity and reproducibility in the structural features (*e.g.*, morphology, pore structure, particle size distribution, and crystalline phase) of the catalyst produced in different batches. Large-scale preparation of many custom-made heterogeneous catalysts is cumbersome and expensive. The recovery and disposal of deactivated catalysts are also often complex. Moreover, the reactions involving H_2_ or O_2_ require extensive process optimization for satisfactory selectivity and yield of the desired product.^40^ The reactor design, safety systems, and workup procedures for reactions involving gaseous reactants at elevated pressure increase capital and operational expenditures. In this regard, stoichiometric oxidants and reducing agents, especially those in liquid and solid states, are more convenient to handle.^41,42^ The selectivity is often high since they can only react with specific functional groups while keeping the other functional groups intact in the substrate. For example, furfural (FF) can be hydrogenated into furfuryl alcohol (FAL) by catalytic hydrogenation using gaseous H_2_ and a suitable metal-based catalyst.^43^ However, depending on the nature of the catalyst and the reaction conditions, overreduction often leads to side products like 2-methylfuran, 2-methyltetrahydrofuran, and tetrahydrofurfuryl alcohol. However, when a stoichiometric reducing agent like NaBH_4_ is used, FAL is produced as the exclusive product.^44^

**Fig. 1:**
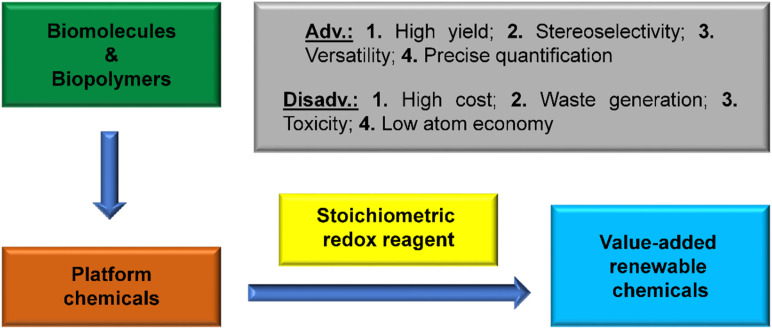
Synthesis value-addition of biomass-derived chemical building blocks by using stoichiometric redox reagents.

This review provides an overview of the oxidation and reduction processes of carbohydrates and the platform chemicals derived from them into organic chemicals of targeted molecular structures, properties, and applications (Fig. 1). Green chemistry metrics, such as atom economy (AE), *E*-factor, and carbon efficiency (CE), have been introduced in various transformations for quantitative comparison with the catalytic processes.^45,46^ The analyses in this review will help identify the prospects and challenges associated with specific stoichiometric reagents for the redox steps. This work will help researchers make an informed decision when designing the synthetic upgrading of carbohydrate-derived renewable chemicals involving one or more redox steps.

## 2. Oxidation reactions

### 2.1 Oxidation by conventional oxidants (*e.g.*, KMnO_4_ and Cr(vi) reagents)

Early literature on producing the oxidized derivatives of HMF involved KMnO_4_ and Cr-based oxidants.^47^ Matsuhisa *et al.* patented the procedure of producing FDCA by oxidizing HMF in an alkaline medium at ambient or near-ambient temperatures.^48^ Cottier *et al.* reported much better yields of 2,5-dimethylfuran (DFF) using various stoichiometric oxidants under ultrasonic irradiation.^49^ The K_2_Cr_2_O_7_–DMSO oxidant system afforded a 58% yield of DFF from HMF after performing the reaction for 5 h at 100 °C. Interestingly, the reaction was completed within 30 min at RT, and the yield of DFF improved remarkably to 75%. The authors also explored the effect of ultrasonication on other catalyst systems. Trimethylammonium chlorochromate was used as the oxidant supported on molecular sieves, affording a 57% yield of DFF in dichloromethane solvent, which increased to 72% under ultrasonication. McDermott and Stockman reported a 65% yield of DFF by oxidizing HMF with pyridinium chlorochromate in dichloromethane solvent, which was then used as the intermediate for synthesizing trioxadispiroketals.^50^ Chaubey *et al.* reported the synthesis of 2-furoic acid (2-FA) by oxidizing FF using quinolinium dichromate in sulfuric acid.^51^ The authors studied the reaction kinetics, and by performing the reaction at 40 °C for 48 h, an 85–90% yield of 2-FA was obtained. A major issue of using KMnO_4_ and Cr(vi) reagents is the toxic metal salts at the end of the reaction. KMnO_4_ forms Mn(ii) salts in the acidic medium, whereas MnO_2_ is produced in the neutral and mildly basic medium. The Cr(vi) reagents are reduced to Cr(iii) salts. The treatment of organic wastes containing these metal salts is rather complicated, especially at larger scales.

### 2.2 Oxidation by nitric acid and nitrates

Nitric acid and various metal nitrates are powerful yet selective oxidants for various biorefinery processes. For example, oxidation of glucose by nitric acid is the primary commercial process for glucaric acid (GRA).^52^ The primary concern of this industrial synthesizing GRA is the large amount of nitric acid left and the release of polluting byproducts (*e.g.*, NO_2_). The separation and purification of GRA from leftover nitric acid is cumbersome. Rivertop Renewables modified this technology by conducting the reaction under an atmosphere of oxygen in a closed reaction flask. The process allowed using a lesser quantity of nitric acid by reoxidizing the NO_2_ into nitric acid.^8^ This is a classic example where a relatively polluting stoichiometric oxidant is used for making a biorenewable product because of the simplicity and scalability of the process. Furanic platform chemicals have also been oxidized into high-value compounds using nitric acid or metal nitrates. Metal nitrates are also used as catalysts for the aerobic oxidation of various bio-derived platform chemicals, but these studies are not discussed in this review for obvious reasons.^53^ Van Reijendam *et al.* reported the oxidation of HMF into DFF by lead tetraacetate, albeit of low yield.^54^ Ai *et al.* have recently reported the oxidation of a relatively concentrated solution of HMF (12.5 wt%) into DFF using nitric acid as the oxidant and solvent, affording up to an 83.2% yield of DFF under optimized conditions (59.5 wt% HNO_3_, 50–60 °C, 7 min).^55^ The authors reported that DFF can be further oxidized into FDCA by increasing the reaction temperature. Brasholz *et al.* reported the production of 5-(chloromethyl)furfural (CMF), 5-(hydroxymethyl)furfural (HMF), and levulinic acid (LA) from fructose under biphasic continuous flow processing. CMF was transformed into various derivatives using stoichiometric oxidizing and reducing agents. For example, CMF was oxidized with 69% aq. HNO_3_ to form FDCA in a 59% yield under optimized conditions (80 °C, 24 h).^56^ Mehdi *et al.* reported the oxidation of benzyl alcohols into the corresponding benzaldehydes using ceric ammonium nitrate (CAN) as the oxidant in imidazolium-based ionic liquids (ILs).^57^ The process afforded a quantitative yield of DFF from HMF under optimized conditions (100 °C, 6 h).

### 2.3 Oxidation by DMSO

Dimethyl sulfoxide (DMSO) can act as a mild oxidizing agent at elevated temperatures to oxidize benzyl halides into an aryl aldehyde following the Kornblum oxidation mechanism.^58^ For example, HMF or the hydrophobic analogs of HMF (*e.g.*, CMF) can be oxidized into DFF by DMSO at elevated temperatures.^59,60^ DMSO is reduced to dimethyl sulfide (DMS), which leaves the reaction medium as a gaseous byproduct.^61^ DMS can be recovered and oxidized back to DMSO to keep using DMSO as an oxidant in a loop. Fructose has been transformed directly into DFF in the DMSO medium, where DMSO promotes dehydration and also oxidizes the HMF intermediate.^62^ The reaction is promoted by a halide ion (*e.g.*, chloride, bromide) that forms 5-(halomethyl)furfural as a transient intermediate before it gets converted into DFF. Kolykhalov *et al.* reported the dehydration of fructose to HMF and the one-pot synthesis of DFF using DMSO as the reaction medium and bromide (*e.g.*, NaBr) as an additive oxidation.^60^ The authors demonstrated that when the reaction temperature was kept at 120 °C, the combination of DMSO and bromide assisted in the dehydration reaction alone, producing HMF in up to 86% yield. Raising the temperature to 150 °C allowed HMF to oxidize into DFF by going through 5-(bromomethyl)furfural (BMF) as the transient intermediate. Under optimized conditions (150 °C, 16 h), a 67% yield of DFF was obtained starting from fructose.

**Scheme 1:**
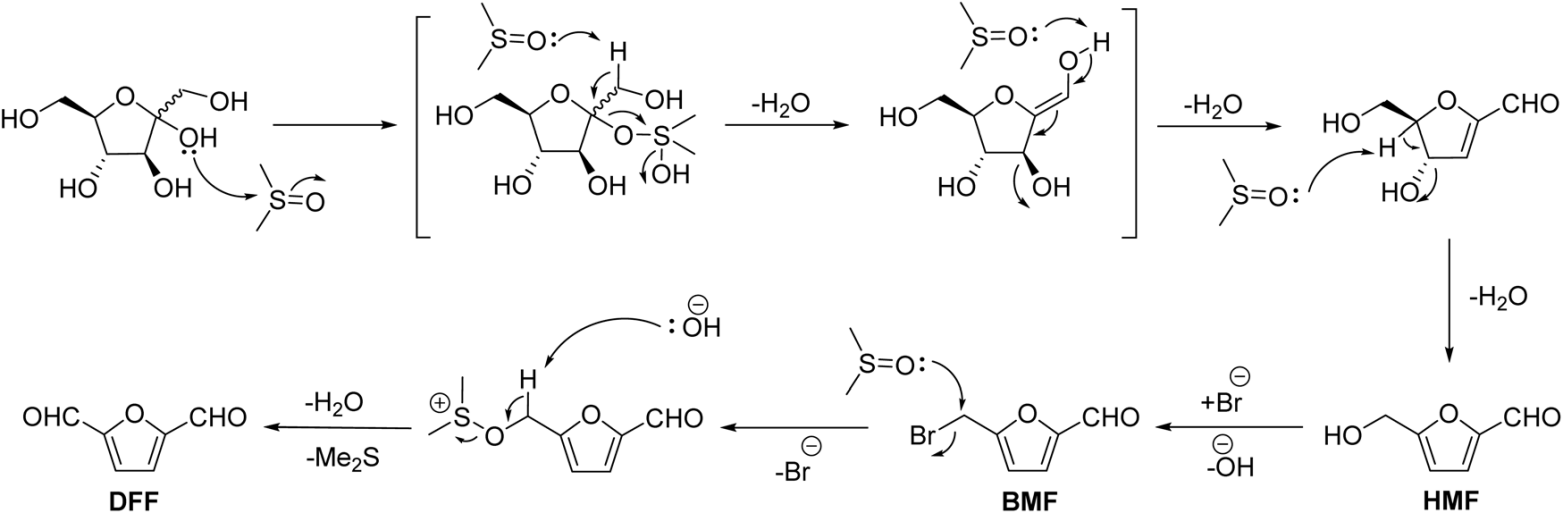
Dehydration of fructose into HMF catalyzed by DMSO, and Kornblum oxidation of HMF by DMSO using bromide ion as the catalyst.

As evident from the mechanistic details shown in Scheme 1, the halide ions significantly influence the Kornblum oxidation, involving DMSO as the oxidant. DMSO cannot substitute the hydroxyl group in HMF; a better-leaving group (*e.g.*, Cl, Br) is required.^59,60,63,64^ Therefore, CMF or BMF works better than HMF, and the latter requires a halide additive to be oxidized into DFF. The significant challenges in this process include slow kinetics, even at relatively high temperatures, which require several hours to complete. The isolation and purification of products from polar, high-boiling DMSO is also challenging. Moreover, DMS must be recovered and oxidized to DMSO to implement this process at scale. A new report by Pang *et al.* shows how DMSO can be used as an oxidant in pressurized air to form DFF from HMF without requiring assistance from the halide ion.^65^ In this case, the challenges in recovering the halide salts from the polar reaction medium have been sorted. While the reaction using DMSO as the stoichiometric oxidant is promising due to its simplicity, exposure to DMSO and DMS can be problematic from health and environmental perspectives. The aerial oxidation of HMF into DMF has the AE of 87.3%, whereas the AE for the transformation involving DMSO is 60.8%.

### 2.4 Oxidation by bromine and iodine

**Scheme 2:**
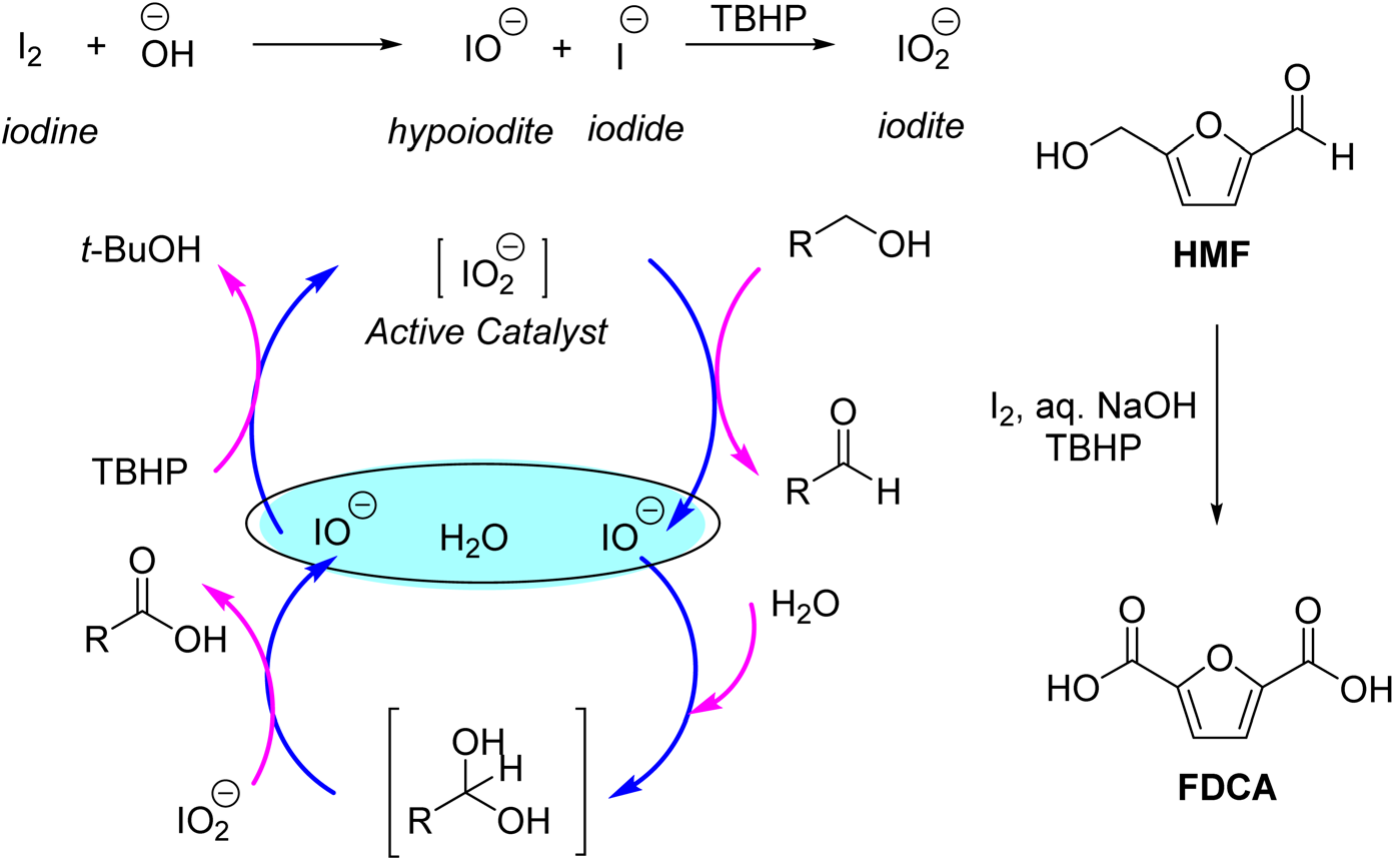
Oxidation of furaldehydes to carboxylic acid by iodite species formed by I_2_ and TBHP in the alkaline medium.

Molecular iodine is a mild and selectivity oxidizing agent used in stoichiometric quantity. In many cases, I_2_ is coupled with a terminal oxidant. In such cases, I_2_ oxidizes the substrate and reduces itself to iodide, reoxidizing into I_2_ by the terminal oxidant. Iodine in the alkaline medium produces more powerful oxidizing species, such as hypoiodite and iodite. Hazra *et al.* reported the oxidation of various benzylic alcohols and aromatic aldehydes into the corresponding carboxylic acids by using a combination of molecular iodine in an alkaline medium and TBHP as the terminal oxidant. Iodite species was proposed to be the active oxidant that oxidized the alcohols and aldehydes into carboxylic acid, itself reduced to the hypoiodite ion. The hypoiodite ion is then reoxidized to iodite by TBHP (Scheme 2).^66^ Therefore, in the true sense, iodine acted as the co-oxidant used in catalytic amounts, whereas TBHC was the terminal oxidant used in an equivalent quantity. The authors reported that neither I_2_ nor TBHP alone afforded any FDCA. The authors reported a 77% yield of 2-FA from FF and a 54% yield of FDCA from HMF, respectively. Dhers *et al.* reported the oxidation of HMF into DFF using Dess–Martin periodinane as the stoichiometric oxidant.^67^ The reaction proceeded at room temperature in dry dichloromethane and afforded an 81% isolated yield of DFF.

Kawasumi *et al.* exploited the iodoform reaction cleverly to synthesize succinic acid (SA) starting from carbohydrate-derived LA.^68^ The reaction involved oxidizing the methyl group adjacent to the ketone functionality in LA using equivalent quality of I_2_ and potassium *tert*-butoxide (*t*-BuOK) as the base. The authors claimed that the *in situ* generated *t*-BuOI acted as the selective iodinating agent for making iodoform from LA. The reaction was then acidified and treated with H_2_O_2_ to liberate I_2_. Iodine was then extracted in dichloromethane, and SA was isolated from the aqueous medium. Iodine can be recovered in the acidic medium by aerial oxidation of iodide. Iodine is then isolated by solvent extraction and then purified by sublimation. Grgur *et al.* reported glucose oxidation into gluconic acid (GCA) using Br_2_ as the stoichiometric oxidant in alkaline water.^69^ The reaction was slow at pH < 8 but accelerated as the pH increased.

### 2.5 Oxidation by bleach

Household bleach (*i.e.*, aq. NaOCl) and other hypochlorites (*e.g.*, Ca(OCl)_2_) are routinely used as inexpensive and selective stoichiometric oxidants for various functional group transformations. Dalcanale and Montanari reported the oxidation of FF into 2-FA by combining NaClO_2_ and AQHP in the acetonitrile–water medium.^70^ The reaction was performed using NaH_2_PO_4_ as the buffer, which produced HClO_2_ as the active oxidant. HMF has been oxidized to DFF using NaOCl as the oxidant in the presence of a Mn(iii)-salen complex as the catalyst.^71^ Recently, HMF has been oxidized into FDCA using NaOCl as the oxidant in the presence of TEMPO as the catalyst.^72^ However, these catalytic transformations are not in the scope of this review.

### 2.6 Organic hypochlorites (*e.g.*, TBHC)

**Scheme 3:**
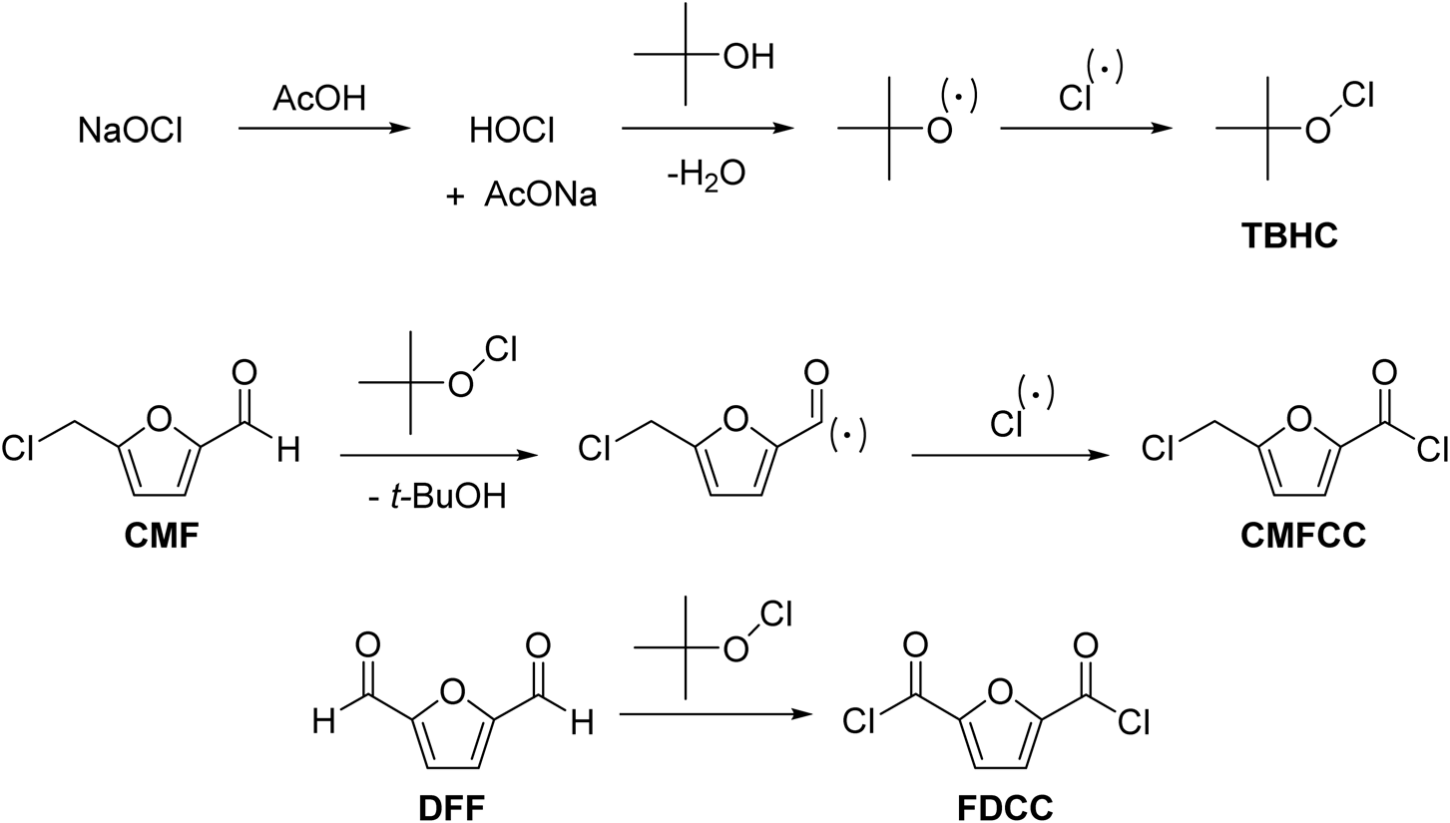
Synthesis of TBHC and its use for oxidizing biorenewable furaldehydes.

Organic hypochlorites are produced by reacting alcohols with chlorine gas or hypochlorous acid. However, most organic hypochlorites are unstable enough to be used as reagents in synthetic processes, except for the rare exception of *tert*-butyl hypochlorite (TBHC). TBHC can be prepared by reacting *tert*-butanol with sodium hypochlorite solution by adjusting the pH with glacial acetic acid.^73^ TBHC has been used as a mild and selective oxidant for oxidizing CMF into 5-(chloromethyl)-2-furancarbonyl chloride (CMFCC) (Scheme 3).^74^ CMFCC has two electrophilic centers (*i.e.*, chloromethyl and carbonyl chloride), and they can be reacted selectively under suitable reaction conditions to form numerous high-value products. For example, CMFCC can react with alcohols (*e.g.*, ethanol) under ambient temperature, where only the carbonyl chloride group reacts and forms esters. The chloromethyl group reacts with alcohols only at elevated temperatures. Similarly, DFF can be reacted with TBHC to form 2,5-furandicarbonyl chloride (FDCC), an organic soluble monomer that can be transformed into polyesters like poly(ethylene 2,5-furandicarboxylate) and other sustainable polymers.^75,76^ The AE of the one-step DFF to FDCC transformation using TBHC is 56.6%. For comparison, the overall AE of the two-step transformation of DFF to FDCC by aerial oxidation of DFF to FDCA followed by reacting the latter with SOCl_2_, is calculated as 48.9%. TBHC is transformed back to *tert*-butanol, which can be conveniently recycled by distillation and reused for preparing fresh TBHC. Therefore, stoichiometric oxidation with TBHC can be made scalable and process economic. When organic SRRs are used, their mechanistic pathway must be studied in detail. Such explorations will assist in minimizing side product formation *via* alternative reaction pathways (*e.g.*, ionic *vs.* radical mechanism). The choice of solvent is also crucial since the radical species may react with the solvent and lead to undesired side products.

### 2.7 Peroxides and peracids

**Scheme 4:**
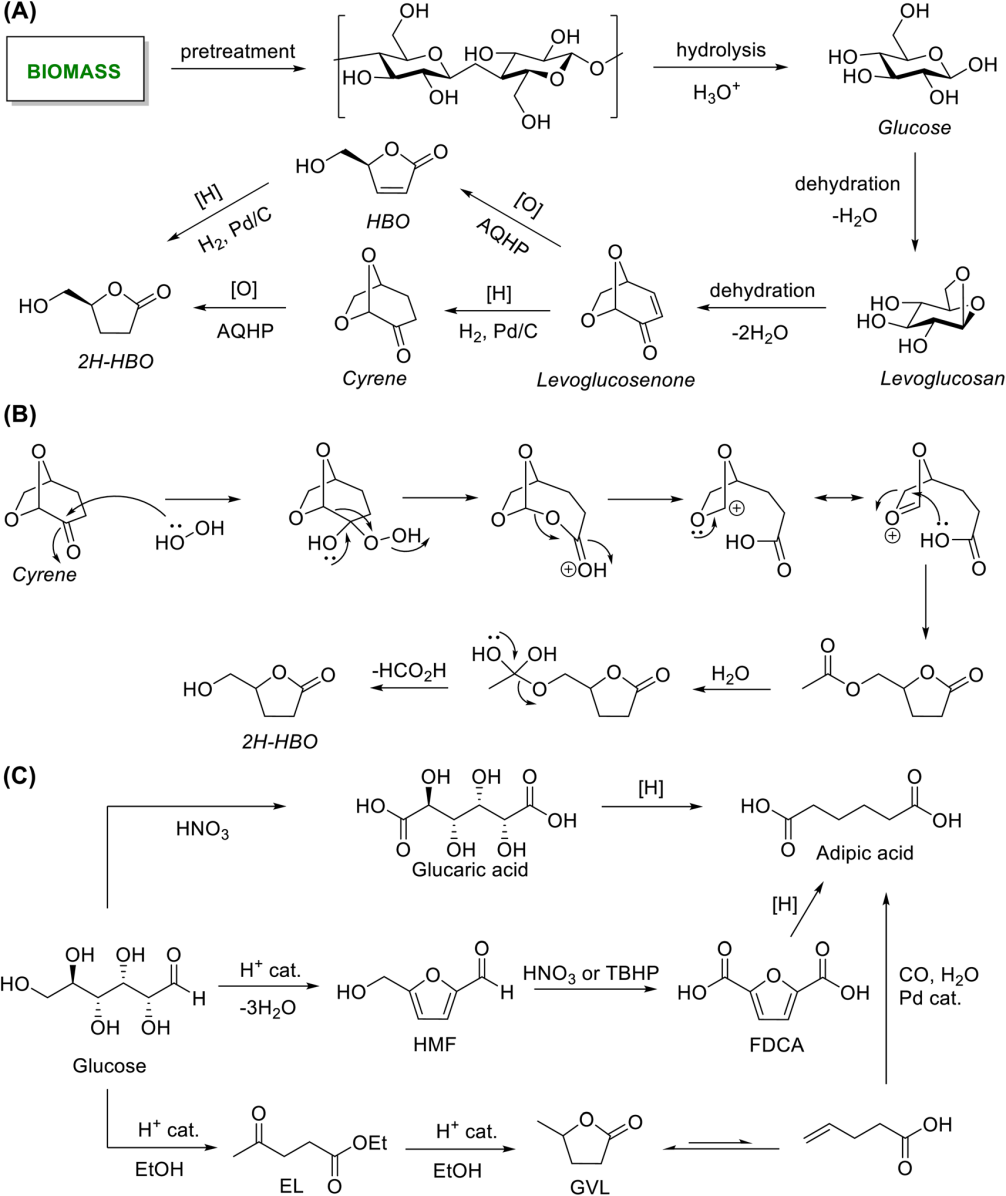
(A) Schematic of the synthesis of HBO and 2H-HBO from biomass, and (B) mechanism of the Baeyer–Villiger oxidation of LGO into HBO and 2H-HBO by AQHP, and (C) synthetic routes to ADA involving SRRs and catalytic redox reactions.

Aqueous hydrogen peroxide (AQHP) is a promising terminal oxidant since it has high activity, selectivity, storage stability, and commercial availability in bulk quantities.^77^ Moreover, AQHP is commercially produced by the catalytic anthraquinone oxidation process using air as the innocuous oxidant.^78^ Even though aqueous hydrogen peroxide (AQHP) is used as the terminal oxidant in many instances, it is routinely used in the presence of a homogeneous or heterogeneous catalyst.^42,79^ Depending on the equivalents of AQHP used in the process and the active oxidant species present in the reaction mixture, it is often debatable whether AQHP can be categorized as a stoichiometric oxidant in these processes. Comprehensive reviews of the catalytic oxidation of biomolecules using AQHP as the oxidant are available.^77,80^ Attempts have been made to produce H_2_O_2_ when and where needed to minimize energy, materials, costs, and logistical complications associated with its transportation and storage. Photochemically and electrochemically generated H_2_O_2_ in the reaction medium *in situ* for the oxidation of biomolecules have received particular interest in this regard.^81^ Attempts have also been made to produce H_2_O_2_ by greener processes, such as the catalytic reduction of atmospheric O_2_.^82^ However, only a handful of studies have attempted using AQHP as the oxidant without a catalyst, possibly because of poor product selectivity. However, the scalability and process economics can be commercially appealing if AQHP shows good activity and selectivity for specific transformations under moderate reaction conditions without requiring a catalyst or organic solvent. For example, Ayoub *et al.* reported a catalyst-free oxidation of FF in AQHP using high-frequency ultrasound (HFUS), leading to a gram-scale synthesis of maleic acid (MA) in good selectivity and yield.^83^ The use of HFUS also accelerated the reaction kinetics at lower temperatures, improving the energy efficiency. Bonneau *et al.* reported the catalyst- and solvent-free oxidation of cellulose-derived levoglucosenone (LGO) and dihydrolevoglucosenone (cyrene) into (*S*)-γ-hydroxymethyl-α,β-butenolide (HBO) and (*S*)-γ-hydroxymethyl-α,β-butyrolactone (2H-HBO), respectively, using AQHP.^84^ The mechanism followed the Baeyer–Villiger mechanism and afforded the enantiopure products in satisfactory isolated yields (Scheme 4). The high-yielding (72%) process allowed for synthesizing enantiopure HBO and 2H-HBO at the kilo scale. The convenient access to these functionally dense molecules from LGO as a chiral chemical platform can provide sustainable access to fine chemicals (*e.g.*, drugs, pheromones, flavors, and fragrances).^85,86^ Mao reported the catalyst-free oxidation of glucose to GCA in AQHP.^87^ The pH of the medium decreased as the reaction progressed due to an increasing amount of GCA produced in the reaction mixture. Under optimized conditions (80 °C, 12% AQHP, 70 min), a 79% yield of GCA was obtained, where the mass balance was unreacted glucose. AQHP is commercially available as a 30–35 wt% solution for chemical synthesis and other purposes. Higher concentrations are also available but used much less frequently. Since H_2_O_2_ slowly decomposes into water and O_2_ even under ambient conditions, higher concentrations of H_2_O_2_ increase the risk of explosion during transportation and storage. Moreover, high concentration of H_2_O_2_ is corrosive and a health hazard.

Organic peroxides have received much interest in sustainable redox reactions since they avoid aqueous medium, and the water-sensitive products are easier to isolate from the organic extractant. *tert*-Butyl hydroperoxide (TBHP) is an organic peroxide that is routinely used as an oxidant, whose reactivity is assigned to the high stability of the radicals (*e.g.*, *t*-BuO˙, *t*-BuOO˙) produced during the mechanistic pathway. Liu and Zhang have recently reported an Au/CeO_2_ catalyst for the fast, high-yielding oxidation of HMF into FDCA using TBHP as the oxidant in the alkaline medium. A 95% selectivity towards FDCA was achieved at the quantitative conversion of HMF (*i.e.*, 95% yield of FDCA) within 30 s at 90 °C.^88^ Adipic acid (ADA) is a commercially important monomer for synthesizing high-volume polymers like nylon-6,6. Significant attempts have been made to produce ADA renewably from various biomass components.^89^ Sugars like glucose can be transformed into ADA through various intermediates, such as GCA, FDCA, GVL, and even 2-FA (Scheme 4C).^90,91^ Even though catalytic steps are given more importance, one or more steps in the synthetic pathway can use SRR. For example, the HMF to FDCA transformation or the glucose to GRA transformation can use stoichiometric oxidants like nitric acid, and then they are catalytically hydrogenated to ADA.

**Scheme 5:**
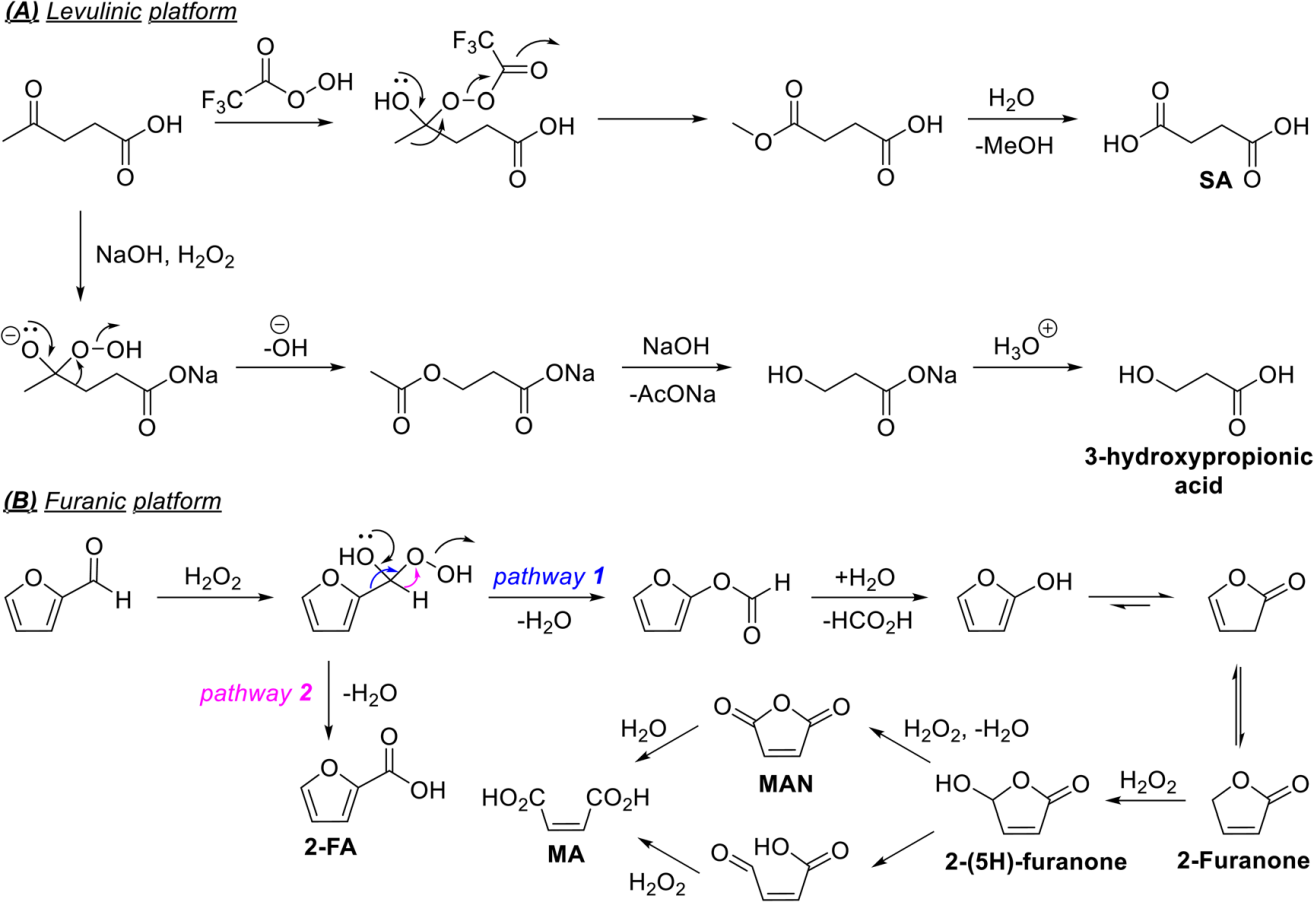
(A) Oxidation of furanic and levulinic platform chemicals by peracids, (B) catalyst-free oxidation of furfural by aqueous H_2_O_2_, and (C) synthesis of ADA from glucose-derived chemical intermediates.

TBHC has been used as an oxidant in the presence of a metal salt or metal-free salt catalyst to form 2-furoic anhydride directly from FF.^92,93^ Organic peracids show remarkable selectivity as stoichiometric oxidants for oxidizing biomolecules directly or the platform chemicals derived from them. These peracids are typically produced *in situ* by reacting AQHP with carboxylic acids. For example, performic acid and peracetic acid can be made by reacting concentrated AQHP (≥30%) with concentrated FA and glacial acetic acid, respectively. The peracids react with 2-furaldehydes by Baeyer–Villiger oxidation reaction, forming a range of valuable products. The selectivity of these products can be manipulated by optimizing the reaction conditions. Mascal *et al.* reported the combination of trifluoroacetic acid (TFA) as the solvent and AQHP as the oxidant for transforming LA into succinic acid (SA).^94^ The reaction formed trifluoroperacetic acid (TFPA) as the active oxidant in the Baeyer–Villiger type oxidation (Scheme 5A). Interestingly, the selectivity was altered by performing the reaction under a strongly alkaline medium, where 3-hydroxypropionic acid was produced as the major product *via* a peroxide intermediate.^95^*m*-Chloroperbenzoic acid was used as the stoichiometric oxidant for the oxidation of cellulose-derived LGO.^96^ The catalyst-free oxidation of FF by AQHP following the Baeyer–Villiger mechanism leads to multiple oxidized products, but the selectivity towards MA is often high (Scheme 5B). Other important oxidized products produced during the mechanistic pathway include maleic anhydride (MAN), 2-furanone, and 2(5*H*)-furanone. The combination of TFA and AQHP, where TFPA is produced as an active oxidant, afforded 2(5*H*)-furanone in a 52% isolated yield under mild conditions (RT, 1 h).^97^ The peracids decompose to the corresponding carboxylic acids during the Baeyer–Villiger oxidation reaction, which can then be recovered and recycled. However, performic acid is relatively fragile and decomposes into CO_2_.^98^

### 2.8 Oxidation by amine oxide

**Scheme 6:**
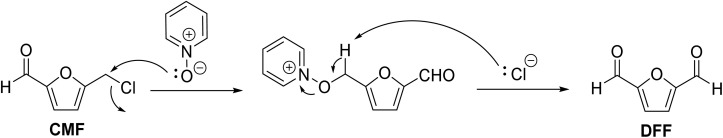
Oxidation of CMF to DFF by pyridine *N*-oxide as the oxidant.

Vicente *et al.* reported that the combination of pyridine *N*-oxide as a stoichiometric oxidant, Cu(OTf)_2_ as a promoter, and acetonitrile as the solvent led to a 54% yield of DFF from CMF under microwave irradiation (MWI) (160 °C, 5 min).^99^ The authors also explored other oxidants for the transformation, such as H_2_O_2_, NaIO_4_, NaBO_3_, and oxone. However, other oxidants afforded only a trace yield (<10%) of DFF. The oxidation process follows the Kornblum oxidation mechanism (Scheme 6). The process gives back pyridine after the oxidation step, which can then be recovered and oxidized to fresh PNO.

## 3. Reduction reactions

### 3.1 Reduction by boranes and borohydrides (*e.g.*, NaBH_4_)

Timko and Cram reported the synthesis of BHMF in a 92% yield by the reduction of HMF with NaBH_4_ as early as 1974.^100^ Brasholz *et al.* reported the reduction of CMF to (5-methylfuran-2-yl)methanol in a 73% yield using NaBH_4_ as the reducing agent.^56^ A symmetric diamine was produced by reacting CMF with benzylamine and then reducing the imine intermediate with NaBH(OAc)_3_ in the acetic acid medium. Several research groups attempted the selective reduction of aldehydes and ketones using NaBH_4_ as the reducing agent, where FF was explored as one of the substrates.^101–103^ FAL were obtained in most of these studies. Sodium cyanoborohydride reduced EL in an acidic medium (pH = 2), affording a 61.7% yield of GVL.^104^ Zhang *et al.* reported the reduction of EL by NaBH_4_ in methanol, affording a 94% yield of GVL.^105^ The byproducts in NaBH_4_ reductions include H_2_, borane, and sodium metaborate (NaBO_2_) due to the reaction of NaBH_4_ with protic solvents (*e.g.*, methanol). The decomposition of NaBH_4_ can be minimized by lowering the reaction temperature, choosing an appropriate solvent, avoiding moisture, and adding a suitable additive (*e.g.*, NaOMe). If the reaction is quenched with mineral acid, it forms sodium salt and boric acid. Interestingly, in earlier literature, LGO was reduced to cyrene by hydride-based stoichiometric reducing agents, such as NaBH_4_ and LiAlH_4_.^96^ Shi *et al.* reported an interesting work using ammonia-borane (AB) as the selective stoichiometric reducing agent for converting FF into FAL in the aqueous medium.^106^ Under optimized conditions (RT, 8 min), a 90% yield of FAL was obtained. Ketones like cyclopentanone required longer duration (2.5 h) but also afforded excellent yield of cyclopentanol. Zhao *et al.* reported using AB as the stoichiometric reagent for the selective reduction of biomass-derived platform chemicals into fine chemicals.^107^ The catalyst-free reactions were performed at ambient temperature using water or methanol as the solvent. For example, ethyl levulinate (EL) was partially reduced to γ-valerolactone (GVL) in a 95% yield. Fructose was directly transformed into GVL in a 60% yield using a combination of Amberlyst-15 as the acid catalyst and AB as the reducing agent. FF and HMF were reduced to FAL and BHMF, respectively, in near quantitative yields by an equivalent amount of AB (RT, 0.5 h, methanol). Moreover, EL was transformed into 5-methyl pyrrolidinone, a renewable organic solvent, by reductive amination in ammoniacal methanol. AB is more atom-economic and eco-friendly than metal-based reducing agents. Future research should focus on using AB and similar systems for various organic transformations and sustainable chemistry.^108^ The byproducts in the reaction include borates, ammonia, and H_2_ gas.

### 3.2 Reduction by hydrogen donor molecules (*e.g.*, formates)

Catalytic hydrogenation uses gaseous hydrogen as the terminal reducing agent in the presence of a suitable metal-based catalyst. On the other hand, a catalytic transfer hydrogenation (CTH) process uses the equivalent amount of some hydrogen donor molecule (HDM) that produces H_2_ during the mechanistic pathway, which immediately reacts with the substrate and forms the reduced product and the oxidized version of HDM. When FA or formates are used as HDM in the presence of a metal catalyst (*e.g.*, Pd/C), they get oxidized to CO_2_. Other commonly used HDMs include 2-propanol, 2-butanol, ethanol, cyclohexene, formaldehyde, and biomass-derived polyols.^109–112^ These HDMs form oxidized products, such as secondary alcohols, which oxidize into ketones, and cyclohexene, which forms benzene. The CTH reaction can be performed under an overpressure of H_2_, or the oxidized HDMs can be reduced by a separate catalytic hydrogenation reaction. The CTH reactions often work under milder conditions than catalytic hydrogenation reactions and afford better selectivity. The use of FA and formates as HDM in the biorefinery processes has been reviewed.^35,113^ The CTH process not only saturates the unsaturated molecules by adding H_2_ molecules across the olefinic group, but it can also cleave the carbon–heteroatom bond (*e.g.*, C–O, C–N) by the hydrogenolysis step.^114^

### 3.3 Reduction by metals

**Scheme 7:**
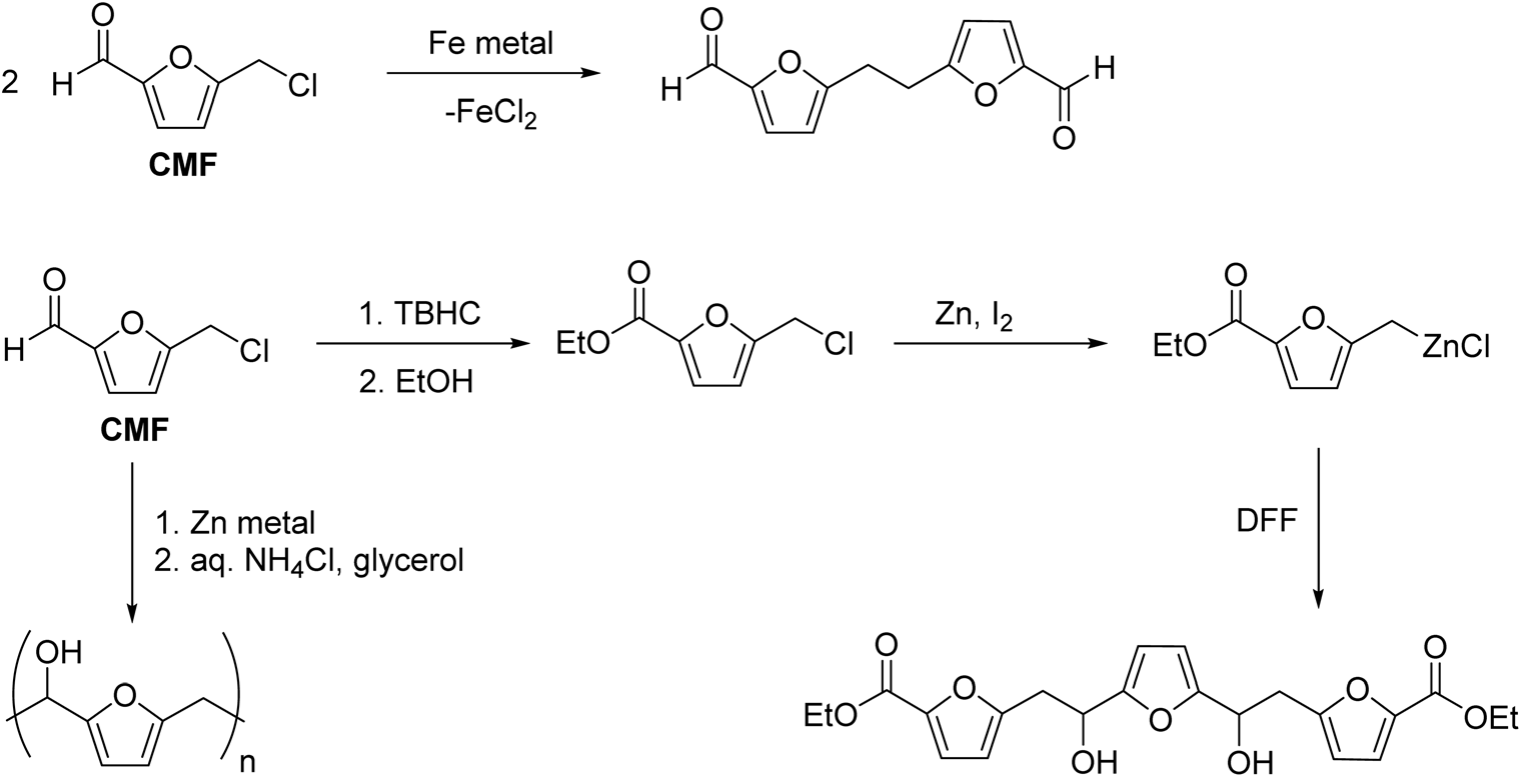
Reactivity and derivative chemistry of CMF with metal as the reducing agent.

Active metals have been used to reduce platform chemicals, such as furaldehydes. CMF was reduced by iron metal by the Wurtz coupling reaction involving the chloromethyl group.^115^ Recently, Mascal *et al.* showed the reactivity of the derivatives of CMF with zinc metal and the synthesis of molecules with extended carbon chain (Scheme 7).^116^ Arslan reported the aqueous Barbier polycondensation reaction of CMF using forming a nucleophilic center on CMF using zinc metal.^117^ Since the polymer has a furan ring and a hydroxyl group, it can be crosslinked using nucleophilic substation or Diels–Alder reaction. The major challenge in using active metals as the electron donor is that they are permanently lost by forming metal salt. Therefore, they should be used in small-scale reactions to produce high-value products, where alternative synthetic strategies are inefficient.

### 3.4 Cannizzaro reaction

**Scheme 8:**
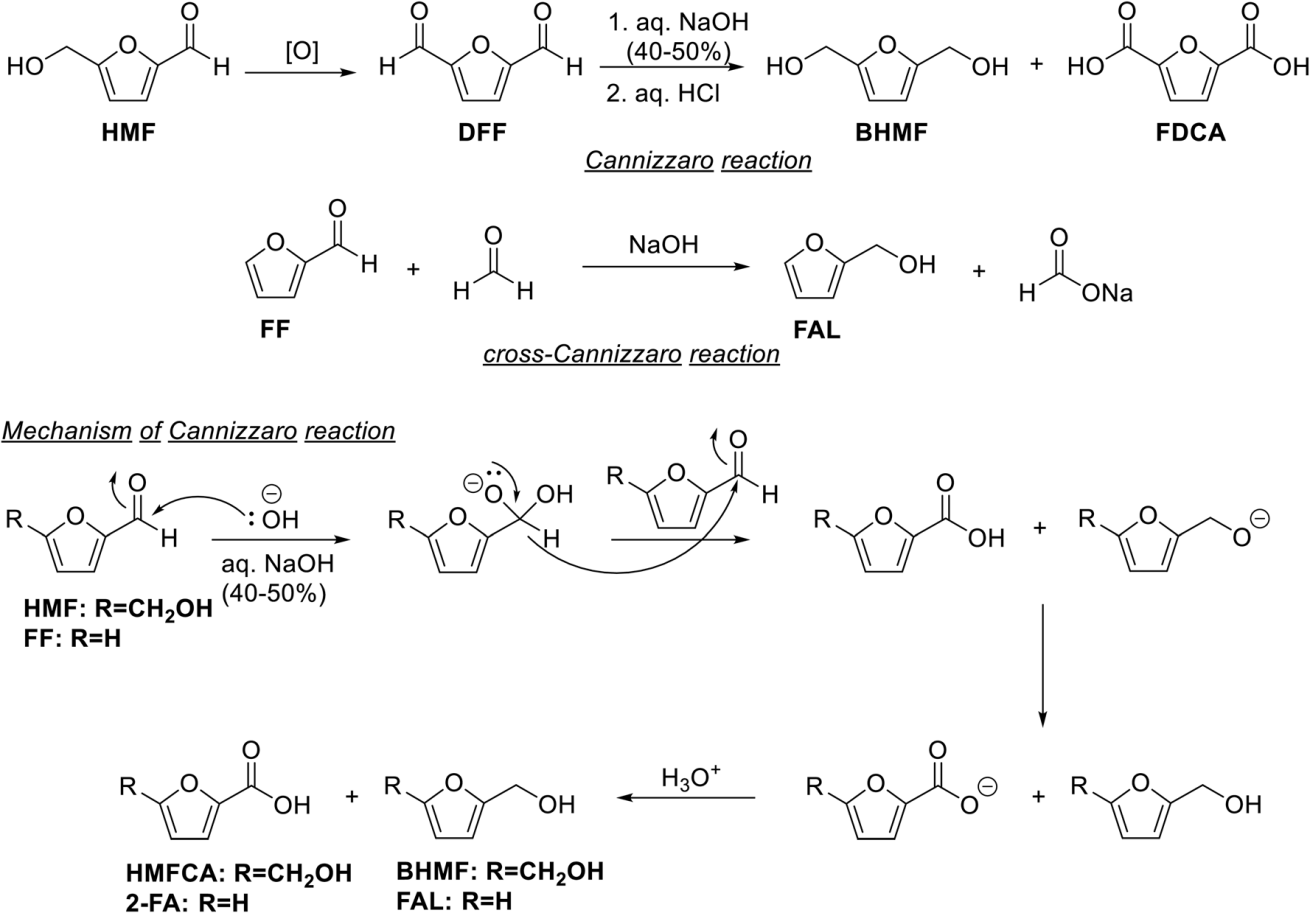
Redox chemistry of carbohydrate-derived furaldehydes using Cannizzaro reaction.

Cannizzaro reaction of aldehydes lacking the α-hydrogen into carboxylic acids and alcohols is a disproportionation reaction.^118^ A suitable aldehyde may be used in excess to manipulate the selectivity towards the desired product. For example, the crossed Cannizzaro reaction between benzaldehyde and formaldehyde produces benzyl alcohol and formates under the reaction conditions.^119^ The reaction works under strongly basic conditions, typically using aqueous alkali. In the case of biorenewable molecules, HMF has been subjected to the Cannizzaro reaction to form 5-(hydroxymethyl)-2-furancarboxylic acid (HMFCA) and 2,5-bis(hydroxymethyl)furan (BHMF).^120,121^ HMFCA and BHMF are important as potential monomers and chemical intermediates for downstream synthetic value-addition pathways. Analogously, when 2,5-diformylfuran (DFF), a partially oxidized product of HMF, is subjected to the Cannizzaro reaction, it produces BHMF and 2,5-furandicarboxylic acid (FDCA) (Scheme 8).^122^ When formaldehyde is used for crossed Cannizzaro with biorenewable aldehydes, the substrate is preferentially reduced to the corresponding alcohol, whereas formaldehyde is oxidized into formate. For example, when FF was subjected to the Cannizzaro reaction under strongly alkaline conditions, 2-FA and FAL were produced in equimolar quantities.^123,124^ Interestingly, 2-FA is currently produced industrially by the Cannizzaro reaction of FF.^123^

However, the crossed Cannizzaro reaction between FF and formaldehyde led to FAL being the major product. Excess formaldehyde led to a further increase in the selectivity towards FAL. Thakuria *et al.* reported the crossed Cannizzaro reaction between paraformaldehyde and FF in the presence of solid NaOH to form FAL (85% yield) under MWI and solvent-free conditions.^125^ When formalin was used instead, basic alumina afforded better results. The Cannizzaro reaction typically has fast kinetics, works under ambient to moderate temperatures, and does not require any external catalyst. Moreover, the products can be separated conveniently since the carboxylic acid product remains salt under the basic medium. In contrast, the alcohol product can be extracted selectively in a suitable organic solvent. If the carboxylic acid and alcohol products are equally valuable, the Cannizzaro reaction can be a valuable synthetic step in a biorefinery setting. The primary concern in this process is using strong alkali conditions, leading to corrosion of reaction parts and producing significant salt streams while isolating the carboxylic acid product. However, process optimizations and using alternative bases can mitigate this issue to a considerable extent.

### 3.5 Reduction by HI

**Scheme 9:**
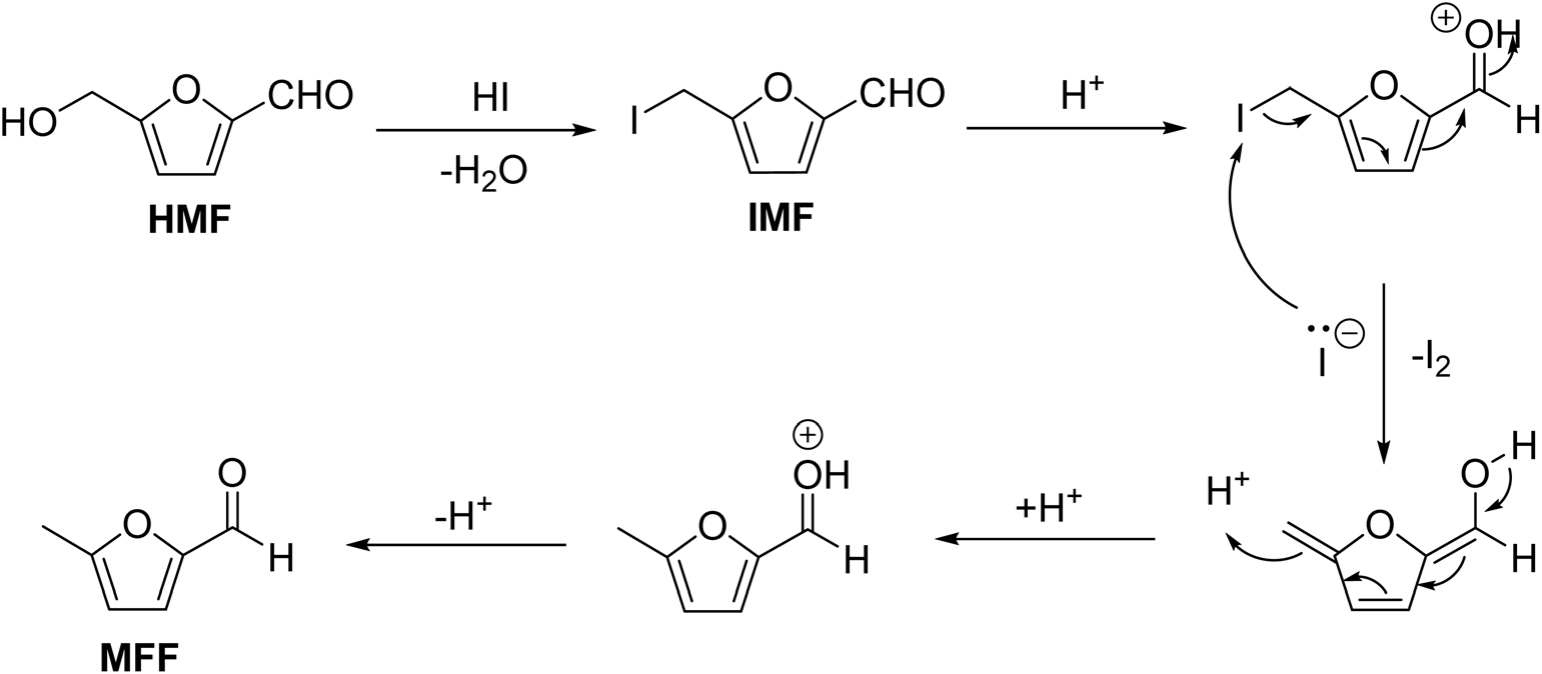
Partial reduction of HMF to MFF by HI.

Hydroiodic acid is a potent reducing agent used in the chemistry of renewables. For example, HMF can be partially reduced to 5-methylfurfural (MFF) by reacting with aqueous HI.^126^ A one-pot process has also been developed, where fructose is first dehydrated into HMF using HI as the acid catalyst, and then it is reduced to MFF using HI as a reducing agent. Hydroiodic acid reduces HMF to MFF through IMF as the intermediate and gets oxidized to I_2_ (Scheme 9). Both ionic and radical mechanisms have been proposed for the transformation. Since HI is strongly corrosive, combining strong Brønsted acids like HCl and iodide salts (*e.g.*, NaI, KI) can generate the same effect as HI.^127^ Since iodine is generated as the oxidized product of HI, a secondary reducing agent (*e.g.*, H_2_) can be used to reduce I_2_ back to HI in the presence of a suitable metal-based catalyst (*e.g.*, RuCl_3_ or Pd/C).^126,127^ FA has also been used as a reducing agent to reduce the I_2_ back to HI. This is an interesting example involving FA as a reducing agent where the metal catalyst requirement is absent.^128^ It is worth noting that the process used MWI. MWI is often preferred over conventional heating due to faster kinetics, energy efficiency, and better selectivity. Even though the processes had the drawback of poor scalability in the initial years, the developments in production-scale MW reactors have significantly improved the prospects of MW-assisted organic synthesis.^129^ Therefore, the scalability and process economics of using HI as a reducing agent can be enhanced, provided it is regenerated in a catalytic loop by reducing the molecular iodine produced as the byproduct.

### 3.6 Reduction by SnCl_2_ and similar reducing agents

Stannous chloride is a mild reducing agent in an acid medium that oxidizes itself into stannic salt. Stannous chloride has been used as a reducing agent in hydrochloric acid to reduce carbohydrate-derived CMF into MFF. According to green chemistry principles, the catalytic redox processes are superior to stoichiometric oxidants or reducing agents. Stoichiometric reagents are more costly than inexpensive terminal oxidants (*e.g.*, O_2_) and reducing agents (*e.g.*, H_2_), and they produce significant waste streams that need to be treated extensively before disposal. However, catalytic redox reactions require the overpressure of gaseous reagents, expensive metal-based catalysts, and higher temperatures (*e.g.*, vapor-phase reactions). Metal-based catalysts, especially those involving rare metals (*e.g.*, Pd, Ru, Pt), are costly, and their production through mining is not sustainable. Moreover, the catalysts eventually deactivate over repeated use, and the metals must be recovered using elaborate chemical processes. Many of the heterogeneous metal catalysts use a supporting material of some sort. The economic and environmental impacts of preparing and disposing of these materials must also be considered. These processes must be considered when calculating the relative advantages of catalytic redox reactions over using the stoichiometric reagents. Selectivity towards the desired product remains a key issue regardless of the type of reagents used. The selectivity for catalytic redox reactions largely depends on the catalyst used. Enormous data and an extensive understanding of the structure–activity relationship of catalysts are required to achieve the desired activity and selectivity. On the other hand, the reactivity of stoichiometric reagents is more predictable. The substrate scope of stoichiometric reagents is also broad since they target the functional groups of the substrate. The catalytic oxidation and reduction reactions require an overpressure, increasing capital and operation expenditures. On the contrary, the stoichiometric oxidants or reducing agents work under milder conditions.

## 4. Conclusion

As discussed in the previous sections, stoichiometric chemical oxidants and reductants continue to be used for the synthetic value addition of biomolecules and biomass-derived platform chemicals. Green oxidants like air and O_2_ have received immense interest, whereas H_2_ gas is the primary reagent for reduction. These reagents are combined with a catalyst, primarily involving one or more metals or their compounds. According to the chemical equations, H_2_ or O_2_ are also stoichiometric reagents, even though they are usually used under overpressure in significant excess. Without a suitable catalyst candidate, these reactions do not proceed or proceed at a sluggish rate. Therefore, these processes are categorically labeled as catalytic processes. Other green oxidants include aqueous H_2_O_2_, whereas green reductants include HDMs like isopropanol. These redox reagents are often used in stoichiometric quantities or slight molar excess. Moreover, these redox reagents work with or without a catalyst candidate. These can be considered SRRs when not using a catalyst candidate. For example, IPA used in the Meerwein–Ponndorf–Verley reduction with a stoichiometric quantity of Al(IPA)_3_ can be viewed as a stoichiometric reducing agent. Even when using a catalyst candidate, if the oxidized derivative of the HDMs is not recycled, they can still be regarded as stoichiometric reagents. The traditional stoichiometric oxidants include Mn and Cr-based reagents, such as MnO_2_, KMnO_4_, K_2_Cr_2_O_7_, CrO_3_, *etc.* Similarly, conventional reducing agents include NaBH_4_, SnCl_2_, and solvated metals. Early literature used these reagents on platform molecules and reported acceptable yields of the desired products. However, significant wastes are produced during the process, and the scalability and economics of the process remain questionable. More recent publications have used much less polluting stoichiometric oxidants and reducing agents. For example, molecular iodine in an alkaline medium is a selective oxidant. Bleach and organic hypochlorites (*e.g.*, TBHC) have received much interest as selective and green oxidants. Nitric and nitrates have also attracted interest as selective oxidants under mild conditions. Moreover, the NO_*x*_ can be reconverted into nitric acid and nitrates by an adjoint catalytic oxidation process. Less polluting stoichiometric reducing agents include various HDMs, such as FA, formates, IPA, 2-butanol, cyclohexene, *etc.* These reagents form significantly less waste compared to traditional oxidizing and reducing agents. Moreover, they function under mild conditions, have high selectivity, and the cost is acceptable depending on the product type. Some transformations allow the disproportionation reaction of the substrate molecule, affording access to both the oxidized and reduced derivative. If both the derivatives have commercial value, these reactions can be employed in the biorefinery setting. For example, the disproportionation of HMF under the Cannizzaro conditions forms BHMF and FDCA in equimolar quantities. The CE for the process is 100% since the process does not produce a carbon-containing byproduct involving a C–C bond-breaking reaction. BHMF and FDCA have immense commercial potential as sustainable monomers of new-generation thermoplastics. However, detailed techno-economic and life-cycle analysis (LCA) of the biorefinery processes involving the SRRs must be compared against the catalytic processes. Organic SRRs are relatively more convenient to use and recycle than metal-based inorganic SRRs. Inorganic metal-free SRRs, such as AB, have significant prospects in sustainable chemistry, and research is expected to intensify in this area. The scalability, process economics, and environmental impacts of redox chemistry using SRRs must be assessed case by case.

## 5. Future perspectives

For obvious advantages, the redox steps in the synthetic pathways have been increasingly catalytic over the past decades. Initially, the catalysts were primarily developed for petrorefinery operations and the synthesis of petrochemicals. However, with the rapid advancement of biorefinery and a global thrust for a circular carbon economy, many of these catalysts have been repurposed for biorefinery processes. A new generation of robust, efficient, and eco-friendly catalysts, including nanocatalysts and single-atom catalysts, have been developed to perform the redox reactions using inexpensive and innocuous reagents (*e.g.*, O_2_, H_2_). Photocatalysts, electrocatalysts, and photoelectrocatalysts are becoming increasingly popular for redox steps with increased efficiency, reduced energy and materials input, and zero to minimal waste formation. However, stoichiometric reagents continue to be used in chemical industries, especially for molecules requiring multistep synthesis. The detailed kinetic and mechanistic studies of the redox reaction have allowed the reaction conditions to be modified for better selectivity, reduced waste generation, and convenient product purification. In some cases, the byproducts were recycled back to the reagent in a closed loop involving catalytic steps. Such combined processes allow for the best combination of the features of stoichiometric and catalytic reagents. SRRs will continue to play significant roles in sustainable synthesis and biorefinery processes. The stoichiometric reagents will be particularly important for synthesizing structurally complex, high-value but relatively low-volume chemicals (*e.g.*, agrochemicals, pharmaceuticals) following a multistep synthesis. For a densely functionalized, structurally delicate molecule, the SRRs often provide a better outcome compared to catalytic redox reactions. The design and synthesis of tailor-made catalysts for such molecules may be expensive and counterintuitive to sustainability. The logical choice of redox reagents should be based on multiple parameters, including the detailed LCA of the reagent, bulk availability, selectivity, process scalability, operational cost, and environmental impact. Transformations allowing disproportionation of the substrate molecules should be explored further since no external redox reagent is required for such processes. However, the use of other reagents in excess, such as bases and acids in the Cannizzaro reaction, should be carefully evaluated to evaluate the relative advantages and disadvantages. Processes that minimize such requirements should be developed without significantly compromising the reaction kinetics and selectivity.

## Abbreviations

ADA: Adipic acid
AB: Ammonia-borane
AQHP: Aqueous hydrogen peroxide
AE: Atom economy
BHMF: 2,5-Bis(hydroxymethyl)furan
BMF: 5-(Bromomethyl)furfural
CE: Carbon efficiency
CTH: Catalytic transfer hydrogenation
CMFCC: 5-(Chloromethyl)-2-furancarbonyl chloride
CMF: 5-(Chloromethyl)furfural
CPN: Cyclopentanone
DFF: 2,5-Diformylfuran
DMF: 2,5-Dimethylfuran
DMS: Dimethyl sulfide
DMSO: Dimethyl sulfoxide
EL: Ethyl levulinate
FMF: 5-(Formyloxymethyl)furfural
FA: Formic acid
FDCC: 2,5-Furandicarbonyl chloride
FDCA: 2,5-Furandicarboxylic acid
FF: Furfural
FAL: Furfuryl alcohol
2-FA: 2-Furoic acid
GRA: Glucaric acid
GCA: Gluconic acid
HFUS: High frequency ultrasound
HDM: Hydrogen donor molecule
HBO: γ-Hydroxymethyl-α,β-butenolide
2H-HBO: γ-Hydroxymethyl-α,β-butyrolactone
HMFCA: 5-(Hydroxymethyl)-2-furancarboxylic acid
HMF: 5-(Hydroxymethyl)furfural
IPA: Isopropanol
LCA: Life-cycle analysis
LA: Levulinic acid
LGO: Levoglucosenone
MA: Maleic acid
MAN: Maleic anhydride
MFF: 5-Methylfurfural
MWI: Microwave irradiation
SRR: Stoichiometric redox reagent
SA: Succinic acid
TEA: Techno-economic analysis
TBHP: *tert*-Butyl hydroperoxide
TBHC: *tert*-Butyl hypochlorite
TFA: Trifluoroacetic acid
TFPA: Trifluoroperacetic acid
GVL: γ-Valerolactone

## Conflicts of interest

The authors declare no competing interest.

## Data Availability

No primary research results, software or code have been included and no new data were generated or analysed as part of this review.
